# A Novel Series of Synthetic Heparin‐Mimetics–Itaconic Acid‐Containing Copolymers for Targeting Tumor Cell Coagulability and Metastasis

**DOI:** 10.1002/mabi.202400633

**Published:** 2025-04-07

**Authors:** Katrin Nekipelov, Abdullah Al Nahain, Sven Otto, Yongbin Xu, Jin‐Ping Li, Natasha Letunica, Simon Collett, Chantal Attard, Paul Monagle, George Vamvounis, John Tsanaktsidis, Vito Ferro, Gerd Bendas

**Affiliations:** ^1^ Department of Pharmacy University of Bonn An der Immenburg 4 53121 Bonn Germany; ^2^ School of Chemistry and Molecular Bioscience University of Queensland Brisbane Queensland 4072 Australia; ^3^ Department of Medical Biochemistry and Microbiology The Biomedical Center University of Uppsala Uppsala 75123 Sweden; ^4^ Haematology Research Murdoch Children's Research Institute The Department of Paediatrics University of Melbourne Department of Clinical Haematology Royal Children's Hospital Parkville Victoria 3052 Australia; ^5^ College of Science and Engineering James Cook University Townsville Queensland 4811 Australia; ^6^ CSIRO Manufacturing Research Way Clayton Victoria 3168 Australia

**Keywords:** cancer, coagulation, heparanase, heparin mimetics, metastasis, PF‐4, platelets

## Abstract

Thromboses are potentially fatal complication in malignant tumor diseases. Today, oral anticoagulants are considered equivalent alternatives to low molecular weight heparin (LMWH) in guideline‐based treatments of cancer‐associated thromboses. Nevertheless, debates on potential antitumorigenic heparin activities beyond anticoagulation are still highly relevant. However, disclosure of heparin targeted activities is complicated by the heterogeneous structure of this glycosaminoglycan of natural origin. Therefore, synthetic polymers appear promising as heparin mimetics to interfere with different steps in tumor metastatic spread. Here, the synthesis of noncarbohydrate copolymers of itaconic acid is described with either potassium‐3‐sulfopropylmethacrylate (SPMA), sodium 4‐styrenesulfonate (SS), or 2‐acrylamido‐2‐methyl‐1‐propanesulfonate (AMPS) via reversible addition‐fragmentation‐chain‐transfer (RAFT) polymerization. The copolymers, characterized by GPC, display high efficiencies to inhibit heparanase enzymatic activity, exceeding the potency of the clinical candidate PG545. The SS‐copolymers (poly(SS‐*co*‐IA)) outperform the other copolymers and LMWH in blocking tumor cell‐induced platelet activation (TCIPA), thus platelet degranulation or aggregation as key issues in metastasis by reducing thrombin formation. The cytotoxicity of poly(SS‐*co*‐IA) is very low. Notably, poly(SS‐*co*‐IA) copolymers displayed a thousand‐fold lower binding affinity to platelet factor‐4 (PF4) than unfractionated heparin (UFH), suggesting a lower risk for HIT II susceptibility. The indicated polymers represent promising heparin mimetics with superior activities in oncology for metastatic control.

## Graphical abstract

Heparin is postulated to possess antitumor properties‐ beyond its anticoagulative activities in oncology, but disclosure of targeted activities is complicated by heparins structural variabilities as natural glycosaminoglycans. Here, synthetic noncarbohydrate polymers are presented with tuneable structures as heparin mimetics in in‐vitro‐approaches for metastatic control. Among different polymer series, poly(SS‐co‐IA) copolymers outperform heparin in blocking heparanase, reducing platelet activation, while minimizing HIT II risk of heparin.

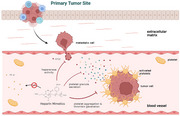

## 1 Introduction

Venous thromboembolism (VTE) is a primary complication in patients with cancer, causing morbidity and severity of disease with a strong impact on mortality.^[^ ^1^ ^]^ Cancer patients possess an up to sevenfold higher risk to suffer from VTE, such as deep venous thrombosis or pulmonary embolism, when compared to the healthy population. The relationship between thrombosis and cancer is mechanistically complex, induced by physical and cellular effects, known for decades.^[^ ^2^ ^]^ Heparin, e.g., low molecular weight heparin (LMWH) has been the primary choice as anticoagulant for prophylaxis and treatment of VTE in oncology for decades. In recent times, treatment paradigms are shifting from LMWH monotherapy to oral anticoagulants, namely direct factor Xa inhibitors (DXI). Updated guidelines thus recommend DXIs for treatment of cancer associated thrombosis (CAT) as an equivalent alternative to LMWH,^[^ ^3^ ^]^ and the decision to select LMWH or DXI is nowadays related to cancer‐ and patient‐associated factors.

Thromboembolism is clearly associated with tumor cell metastasis, which is considered the primary cause of death in cancer.^[^ ^4^ ^]^ Hematogenous metastasis of tumor cells is a highly complex and orchestrated process. In these terms, tumor cells receive multiple benefits by the interaction with host cell populations, such as platelets.^[^ ^5^, ^6^ ^]^ When entering the blood stream, platelets form a dense cloak surrounding the circulating tumor cells protecting them from physical shear stress or attacks by immune cells.^[^ ^6^ ^]^ This interaction, synonymous with activation of platelets by the tumor cells (tumor cell‐induced platelet activation – TCIPA) is based on coagulation, thus thrombin receptor activities.^[^ ^6^ ^]^ Heparins, e.g. LMWHs, have been confirmed to interfere with TCIPA by multiple modes of action, mainly blocking thrombin formation and possessing anti‐adhesive characteristics.^[^ ^7^ ^]^ Based on these facts, a still ongoing and partly controversial discussion exists on whether or how UFH and LMWH can possess anti‐tumorigenic activities beyond, or based on anticoagulant activities. This debate is still triggered by the glycosaminoglycan (GAG) nature of heparin giving rise to multiple interactions of the linear polysulfated polysaccharide structure with various biological mediators or even receptors in a charge mediated manner.^[^ ^8^ ^]^ However, considerations and elucidations of those “pleiotropic” effects of heparin in oncology^[^ ^9^ ^]^ were further complicated by the fact that heparin is a highly heterogeneous natural product with underlying variations in size and glycosidic composition. Although anticancer effects of LMWH have been postulated from clinical findings^[^ ^10^ ^]^ and more recent data, e.g., imply benefit for pancreatic cancer patients when treated with LMWH,^[^ ^11^ ^]^ clear relationships and elucidation of targeted activities of heparin with clinical implications are hard to derive due to the indicated structural peculiarities. Furthermore, UFH and LMWH possess some further drawbacks, such as strict dependency and limitation of the supply chain, a narrow therapeutic window, variations in bioavailability and pharmacokinetics. Furthermore, UFH can cause major adverse events, e.g., heparin induced thrombocytopenia type II (HIT II).^[^ ^12^ ^]^

Consequently, synthetic polymers, mimicking the polyanionic nature of heparin, but possessing narrowly defined structural parameters, such as molecular size, degree of sulfation and dispersity appear promising to elucidate the molecular requirements of heparin in this context, and in a wider perspective, to substitute heparin in medical applications. In these terms, several chemical structures have been introduced as potential heparin mimetics into different preclinical investigations.^[^ ^13^, ^14^ ^]^ Recently, we have generated a series of non‐carbohydrate heparin mimetic polymers with low *Ð* and good MW control produced by the reversible addition‐fragmentation chain transfer (RAFT) polymerization of sulfonated and carboxylated monomers.^[^ ^14^, ^15^, ^16^ ^]^ Homopolymers, prepared from sodium 4‐styrenesulfonate (SS), sodium‐2‐acrylamido‐2‐methyl‐1‐propane sulfonate (AMPS), potassium‐3‐sulfopropyl acrylate (SPA), or potassium‐3‐sulfopropyl methacrylate (SPMA), or their copolymers with acrylic acid (AA) were characterized with respect to their anticoagulant activities.^[^ ^15^ ^]^ Recent data confirm that the copolymers, prepared from SS and AA, poly(SS‐*co*‐AA) appeared most promising with respect to anticoagulation. They also shared some further targeted activities with LMWH in a simulated metastatic context, e.g. blocking adhesion receptors such as selectins and the integrin VLA‐4, or even outperformed LMWH in blocking heparanase.^[^ ^17^ ^]^ These highly promising data on anticoagulant synthetic heparin mimetics prompted us to further modify these copolymers for optimized activity. Since one critical aspect appears to be charge density, we replaced AA, used in the previous series by the more highly charged itaconic acid (IA) as a copolymer constituent with SS, AMPS, or SPMA, thus preparing three novel series of IA‐based copolymers by RAFT polymerization.

Herein we describe the synthesis and physicochemical characteristics of the novel copolymers poly(SS‐*co*‐IA), poly(AMPS‐*co*‐IA), and poly(SPMA‐*co*‐IA). Furthermore, these polymers were characterized with respect to different potential targets in a simulated metastatic process. Notably, all polymers appear highly promising in blocking enzymatic activity of heparanase that is regarded as a target for prevention of tumor metastasis. Furthermore, the SS moiety was confirmed to be essential to mediate anticoagulant activities of the copolymers, and therefore impact coagulation‐dependent targeted activities, such as platelet activation and thrombin formation. Despite selected copolymers of the poly(SS‐*co*‐IA) series clearly outperforming LMWH and partly UFH in these activities, biosensor data confirm that these polymers have a much lower binding affinity to PF4 than UFH, thus possessing a significantly reduced risk to potentially induce HIT II. Consequently, the non‐carbohydrate copolymers show potential as alternatives to commercial heparins with reproducible synthesis and improved efficacy with a reduced side effect profile.

## 2 Results and Discussion

### 2.1 Synthesis of a Novel Series of Itaconic‐Acid Based Heparin –Mimicking Copolymers

**Figure 1:**
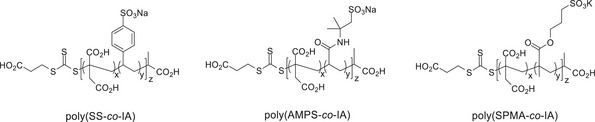
Structures of the itaconic acid copolymers prepared in this study.

Based on our recent experiences to generate non‐carbohydrate‐based heparin mimetics by RAFT polymerization of sulfonated and carboxylated monomers,^[^ ^14^, ^15^, ^16^ ^]^ we followed this approach to form a novel series of linear, negatively charged heparin mimetics based on itaconic acid copolymerized with sodium 4‐styrenesulfonate (SS), sodium‐2‐acrylamido‐2‐methyl‐1‐propane sulfonate (AMPS), or potassium‐3‐sulfopropyl methacrylate (SPMA), resulting in the indicated copolymers poly(SS‐*co*‐IA), poly(AMPS‐*co*‐IA), and poly(SPMA‐*co*‐IA), shown in **Figure** **1**. The copolymers were prepared with targeted MW ranges of either 5, 10, or 20 kDa and in varying proportions of itaconic acid to the indicated sulfonated building blocks. Polymerizations were conducted in water under microwave heating using the water‐soluble RAFT agent BM1429. The polymers were isolated by precipitation from acetone and characterized by ^1^H NMR spectroscopy and GPC (see **Table** **1** and Figure S11, Supporting Information). As can be seen from Table 1, IA copolymers with SS and AMPS all had very low dispersities (1.04–1.09). However, the monomer conversion of IA was typically less than for SS, indicating that IA is less reactive than SS and these copolymers are likely to have longer sections of SS units due to slower incorporation of IA. The *M*_n_ determined by GPC was generally higher than the theoretical *M*_n_ determined by NMR, also indicating an SS‐rich copolymer consisting of long SS sequences within the copolymer chains. Unfortunately, polymerizations with SPMA were not as well controlled, as indicated by the much higher *M*_n_ and dispersities. The GPC analysis also showed some bimodal distributions (Figures S1–S10, Supporting Information), suggesting two distinct polymerisation events consistent with a poorly controlled polymerisation process typical of monomers with very different reactivity ratios. For some copolymers where the conversion of the sulfonated monomer was close to 100% and IA conversion was much lower (e.g., poly(SS‐*co*‐IA) 1:1 10 kDa or poly(SPMA‐*co*‐IA) 1:1 10 kDa), the composition of the chain end formed during the last stages of the polymerization is likely to be particularly rich in IA, since the sulfonated monomer was then almost used up. These structural features may play a role in the observed biological activities (*vide infra*). As seen in our previous studies, the relative content of both monomers in each copolymer is also an important factor for biological activity. We have therefore determined the ratio of the two monomers in each copolymer by NMR spectroscopy, and have expressed this as the sulfonate to carboxylate ratio in Table 1.

**Table 1:** Summary of the monomer conversion (MC), molecular weight, dispersity, product yield and sulfonate to carboxylate ratio for the IA copolymers with SS, AMPS and SPMA for various monomer ratios.

Polymer	MC^a)^[%]		*M*_n_ ^b)^ [kDa]	*M*_n_ ^c)^ [kDa]	*Ð* ^c)^	Yield^d)^ [%]	SO_3_^−^:CO_2_^−^ Ratio^e)^
	X	IA					
Poly(SS‐*co*‐IA) 1:1 5 kDa	100	42	3.9	27.2	1.07	62	1.84
Poly(SS‐*co*‐IA) 1:1 10 kDa	100	58	8.4	34.8	1.05	38	1.62
Poly(SS‐*co*‐IA) 1:1 20 kDa	98	50	15.7	40.2	1.07	20	2.28
Poly(SS‐*co*‐IA) 2:1 5 kDa	100	83	4.9	28.7	1.04	63	1.18
Poly(SS‐*co*‐IA) 2:1 10 kDa	99	81	9.4	35.4	1.05	37	1.04
Poly(SS‐*co*‐IA) 2:1 20 kDa	91	85	17.5	41.9	1.07	41	0.78
Poly(SS‐*co*‐IA) 4:1 5 kDa	100	100	5.0	27.8	1.04	66	0.72
Poly(AMPS‐*co*‐IA) 2:1 10 kDa	100	100	18.0	34.6	1.06	88	0.70
Poly(AMPS‐*co*‐IA) 2:1 20 kDa	90	100	32.7	37.9	1.08	82	0.55
Poly(AMPS‐*co*‐IA) 4:1 10 kDa	100	100	19.9	36.4	1.06	80	0.20
Poly(AMPS‐*co*‐IA) 4:1 20 kDa	100	100	41.7	44.2	1.09	78	0.26
Poly(SPMA‐*co*‐IA) 1:1 5 kDa	100	41	4.1	74.3	1.30	44	1.07
Poly(SPMA‐*co*‐IA) 1:1 10 kDa	100	38	7.9	87.8	1.45	53	1.23
Poly(SPMA‐*co*‐IA) 1:1 20 kDa	99	39	15.6	98.0	1.56	57	1.73
Poly(SPMA‐*co*‐IA) 2:1 5 kDa	100	100	5.2	86.2	1.40	66	0.91
Poly(SPMA‐*co*‐IA) 2:1 10 kDa	100	45	9.0	108.2	1.73	41	0.85
Poly(SPMA‐*co*‐IA) 2:1 20 kDa	100	49	18.0	134.9	1.82	22	0.82
Poly(SPMA‐*co*‐IA) 4:1 5 kDa	100	51	4.4	108.7	1.66	59	0.89
Poly(SPMA‐*co*‐IA) 4:1 10 kDa	99	60	8.7	136.7	2.45	86	1.26
Poly(SPMA‐*co*‐IA) 4:1 20 kDa	99	43	16.8	168.6	2.69	94	0.97

^a)^ As determined by ^1^H NMR spectroscopy;

^b)^ Theoretical molecular weights (see Materials and Methods);

^c)^ Determined by gel permeation chromatography (GPC);

^d)^ Determined gravimetrically;

^e)^ Determined by ^1^H NMR spectroscopy.

### 2.2 Copolymers Inhibit Tumor Cell‐Induced Platelet Granule Release

The generally high coagulability of tumor cells has strong consequences for platelets, the first and dominant host cell fraction that tumor cells encounter when entering the blood system in terms of hematogenous metastasis. As a result, platelets are activated by the tumor cells, forming a platelet cloak around them. Platelet activation is indicated by the release of platelet granules, which contain a multitude of growth factors, chemokines and signaling molecules thus fostering the process of metastatic tumor spread. Heparin is known to inhibit this platelet activation by possessing both anticoagulant and antiadhesive properties.^[^ ^18^ ^]^ Recent data confirm that certain heparin mimetic polymers can attenuate platelet activation depending on their structural composition.^[^ ^17^ ^]^

To follow experimentally the degranulation of activated platelets, and the interference thereof by the heparin mimetic polymers, we analyzed the release of ATP as an indicator of dense granule release from platelets after contacting MDA‐MB‐231 breast cancer cells. ATP was quantified via luminescence measurements. Non‐activated platelets served as a negative control, and platelet activation via thrombin receptors using TRAP‐6 (T6) was regarded as maximal degranulation.

**Figure 2:**
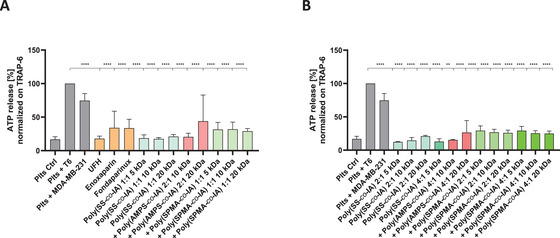
Detection of ATP, released from platelet granules after activation by tumor cells and inhibition of the degranulation by heparin mimetics. A) The data show that the mimetics of the three IA‐based copolymer series are equivalent in potency to LMWH in blocking platelet activation and degranulation, displaying a trend of highest activity for the poly(SS‐*co*‐IA) mimetics. B) Changes in molar ratio of the indicated polymers were well tolerated with respect to maintain inhibitory capacities. Normalization was performed on TRAP‐6 in both graphs.

Comparing the three different copolymers, poly(AMPS‐*co*‐IA), poly(SPMA‐*co*‐IA), and poly(SS‐*co*‐IA), in a 1:1 or in a 2:1 monomer ratio, each at the three different target MW of 5, 10, and 20 kDa applied at 50 µg mL^−1^ clearly indicate that the polymers have a remarkable activity to attenuate the ATP release from platelets with a similar intensity to UFH, exceeding the capacity of LMWH or the Xa‐inhibitor fondaparinux in this context (**Figure** **2**A).

Poly(SS‐*co*‐IA) copolymers displayed a trend to higher inhibitory activities compared with most of the other mimetics. These polymers can also inhibit ATP release if the content of sulfonated monomer and MW is modified, e.g. increased (Figure 2B).

These data indicate that the polymers are able to inhibit degranulation of platelets in terms of TCIPA and thus in a comparable manner like conventional heparin derivatives.

### 2.3 Heparanase Inhibitory Capacity of the Heparin Mimetics

Heparanase (HPSE) is profoundly expressed in human platelets, consequently release of HPSE in terms of platelet activation contributes to the tumor metastasis process. Since HPSE is an established target for treatment of metastatic cancers, attenuation of HPSE release from platelets and furthermore direct inhibition of HPSE endo‐β‐D‐glucuronidase enzymatic activity appear promising approaches using heparin mimetics. Several carbohydrate‐derived heparin mimetic compounds have been evaluated in clinic research as potential HPSE inhibitors.^[^ ^19^ ^]^ Unfortunately, none of the compounds have been approved for use for various reasons. In our previous study we detected the outstanding inhibitory potential of poly(SS‐*co*‐AA) heparin mimetics compared to established HPSE inhibitors.^[^ ^17^ ^]^

**Figure 3:**
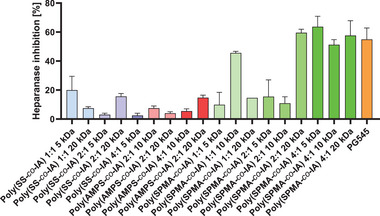
Heparanase inhibition assay. Percentage inhibition of enzymatic activity of HPSE by heparin mimetics at a concentration of 0.15 µg mL^−1^ compared to the established clinical candidate PG545 (0.75 µg mL^−1^). Data indicate an outstanding activity of polymers of the poly(SPMA‐*co*‐IA) series, followed by poly(SS‐*co*‐IA) mimetics.

To further address this issue in terms of the novel copolymers, we examined the capacity of the mimetics of the IA series to block the enzymatic activity of HPSE using a fluorescence‐based assay. All mimetics were compared on a mass basis detecting the percentage HPSE inhibition when using 0.15 µg mL^−1^ of the indicated polymers. Notably, mimetics of the poly(SPMA‐*co*‐IA) series clearly surpass the mimetics of the other two series (**Figure** **3**), showing more than 50% of HPSE inhibitory activity, equivalent to the established HPSE inhibitor PG545^[^ ^20^ ^]^ that was used at a fivefold higher amount. It must be taken into account that all copolymers of this series had very high MWs and high dispersities (1.30–2.69). However, the poly(SS‐*co*‐IA) mimetics, which fit the targeted MW much better than the poly(SPMA‐*co*‐IA) series with low dispersities, also displayed remarkable activity, e.g. showing 10% inhibition for the 20 kDa derivative of poly(SS‐*co*‐IA) 1:1 and slightly higher for poly(SS‐*co*‐IA) 2:1 20 kDa, respectively. Therefore, both indicated copolymers were directly compared with PG545 in another set of heparanase inhibition experiments to derive the molar IC_50_ values, given in **Table** **2**. These data confirm outstanding inhibitory capacities of the copolymers in the nanomolar range clearly exceeding the activity of PG545.

**Table 2:** IC_50_ values of selected compounds for heparanase inhibitory capacities.

Compound	IC_50_ [nм/L]
Poly(SS‐*co*‐IA) 1:1 20kDa	15.5
Poly(SS‐*co*‐IA) 2:1 20 kDa	16.5
PG545	794

Concluding, the high capacity of the novel copolymer mimetics, e.g. the poly(SS‐*co*‐IA) series to interfere with HPSE activities make them promising candidates to interfere with further steps in the metastatic cascade.

### 2.4 Heparin Mimetics Affect Platelet Aggregation Depending on the Copolymer Composition

For a further insight how copolymer structure impacts platelet activation by tumor cells, the copolymers poly(SPMA‐*co*‐IA), and poly(SS‐*co*‐IA), each at a 1:1 monomer ratio and poly(AMPS‐*co*‐IA) in a 2:1 ratio, were selected at a targeted size of 20 kDa to investigate their capacity to attenuate platelet aggregation after tumor cell contact using light transmission aggregometry (LTA). Platelet aggregation might be considered a more sensitive parameter of TCIPA reflecting the activation status of platelets than degranulation. In terms of metastatic spread, aggregation offers protection of tumor cells against shear forces of the blood stream and attacks of immune surveillance. Aiming to detect interference with aggregation, platelets were pre‐incubated with the different copolymers at a concentration of 50 µg mL^−1^ and then activated with MDA‐MB‐231 cells in order to trigger aggregation. Again, thrombin receptor activating peptide TRAP‐6 was used as a positive control for the maximal aggregation of platelets.

**Figure 4:**
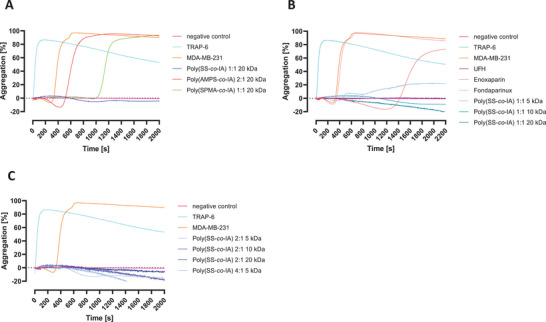
Tumor cell‐induced platelet aggregation and interference by heparin mimetics. A) Comparing the derivatives of the three polymer series at a targeted MW of 20 kDa, only the poly(SS‐*co*‐IA) derivative is able to block aggregation, while the others induce a delay until reaching full aggregation of platelets. B) Comparing poly(SS‐*co*‐IA) mimetics with conventional heparin and anticoagulant underscores the outstanding capacity of the mimetics in blocking TCIPA. C) Changing the monomer ratio has no negative impact on the inhibitory capacity of the poly(SS‐*co*‐IA) mimetics.

Notably, only the copolymer poly(SS‐*co*‐IA) is able to completely inhibit aggregation, while the two other copolymers failed, and induced only a delay until reaching full aggregation (**Figure** **4**A). These findings support the trend seen above in ATP release, which emphasize the role of SS‐moiety in the polymers for blocking platelet activation, previously shown.^[^ ^17^ ^]^ In addition, also the polymers of SPMA and AMPS failed to show any activity in these approaches (data not shown).

Further, the capacities of the poly(SS‐*co*‐IA) polymers to attenuate TCIPA were compared in relation to conventional heparins. Since UFH depresses TCIPA completely, while enoxaparin induces only a delay until reaching full aggregation, and the pentasaccharide fondaparinux failed to show any activity, this implies a certain size dependency for activity. This is also reflected by the poly(SS‐*co*‐IA) polymer series, in which the two polymers with higher MW (targeted MW 10 and 20 kDa, exp. 34.8 and 40.2 kDa, respectively) surpass the activity of the lower MW derivative, which does not depress TCIPA completely (Figure 4B).

To further focus the impact of the content of SS in the copolymer chain, selected copolymers were applied (Figure 4C). It clearly indicates that higher ratios of SS (2:1 or 4:1) to IA are well‐tolerated. In contrast, even by changing the MW or the monomer ratios, no increase in antiaggregative activity could be achieved with the AMPS or SPMA‐based copolymers (Figure S12, Supporting Information) indicating the essential role of the SS moiety for blocking platelet activation.

### 2.5 Poly(SS‐co‐IA) is Superior to the Other Polymers in the Inhibition of Tumor‐Induced Thrombin Generation

**Figure 5:**
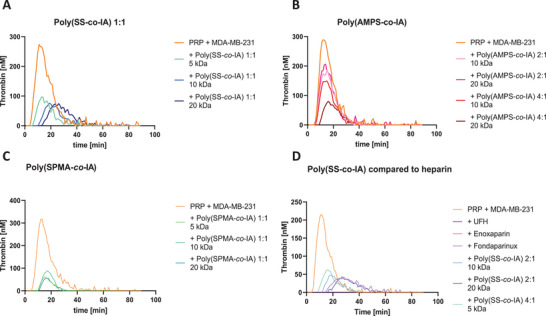
Indicated heparin mimetics inhibit thrombin generation to a different extent. Platelet rich plasma (PRP) was incubated with A) poly(SS‐*co*‐IA), B) poly(AMPS‐*co*‐IA), C) poly(SPMA‐*co*‐IA), and D) poly(SS‐*co*‐IA) compared to heparin at a concentration of 600 µg mL^−1^. Thrombin generation was induced by MDA‐MB‐231 cells and monitored for 90 min. Representative graphs of thrombin generation kinetics are shown.

TCIPA is underlying complex mechanisms, but the activation of the plasmatic coagulation cascade, thus generation of thrombin is considered the initial stimulus in this scenario. Thrombin, once it has been formed initiated by the tissue factor expression of the tumor cells, activates platelets by cleavage of PAR 1, or PAR 4, in addition to multiple other prometastatic effects.^[^ ^21^ ^]^ MDA‐MB‐231 cells express tissue factor, and the impact on TCIPA via the thrombin pathway has recently been confirmed in this context.^[^ ^7^ ^]^ To investigate whether the indicated heparin mimetics are able to interfere with tumor cell‐induced thrombin formation, platelet rich plasma was incubated with the different compounds and the kinetics of thrombin formation after activation with MDA‐MB‐231 cells were recorded. It is clearly evident that poly(SS‐*co*‐IA) 1:1 (**Figure** **5**A) and also (SPMA‐*co*‐IA) 1:1 copolymer series (Figure 5C) (Figure S13, Supporting Information) are superior to the poly(AMPS‐*co*‐IA) mimetics (Figure 5B) – except for the poly(AMPS‐*co*‐IA) 4:1 20 kDa copolymer. To further focus on the structurally well‐defined poly(SS‐*co*‐IA) polymers, they were directly compared to UFH and LMWH (Figure 5D) at increased SS/IA ratios, showing equal efficiency to repress thrombin formation to LMWH.

Structurally, the presence of the SS moiety again appears essential, considering the superior activity of the poly(SS‐*co*‐IA) mimetics in the 1:1 ratio (Figure 5A) compared to the lower activity of, e.g., the mimetics of the poly(AMPS‐*co*‐IA) series (Figure 5B). These findings explain the superior activity of the poly(SS‐*co*‐IA) derivatives in the above assays blocking TCIPA. However, UFH and enoxaparin show the strongest inhibition of thrombin generation represented by a course of the curve close to the x‐axis (Figure 5D). Consequently, the poly(SS‐*co*‐IA) series was focused for the further investigations as potent heparin mimetics to interfere with tumor cell activities.

### 2.6 Confirming Anticoagulant Activities of Poly(SS‐co‐IA) by Detecting Activated Thromboplastin Time

**Figure 6:**
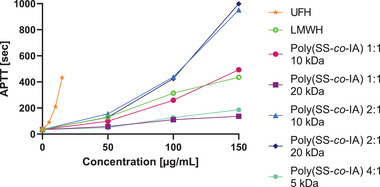
Activated partial thromboplastin time (APTT) for different heparin mimetics of the poly(SS‐*co*‐IA) series at 50, 100, and 150 µg mL^−1^ in pooled normal plasma in comparison to LMWH at this concentration. UFH was used as a further control with the concentrations of 5, 10, and 15 IU mL^−1^.

To further characterize the anticoagulant activities of the poly(SS‐*co*‐IA) copolymer series, the activity of the indicated polymers in normal pooled plasma was assessed at various concentrations (50, 100, and 150 µg mL^−1^) detecting the activated partial thromboplastin time (APTT), as described before.^[^ ^16^ ^]^ All the derivatives prolong the APTT remarkably indicating an anticoagulant activity. There is a clear trend visible that the proportion of the SS moiety affects the activity in a positive way, comparing the derivatives of a targeted MW of 10 and 20 kDa with different copolymer ratios. This effect can be canceled out if the proportion of SS is too high, as can be seen in the polymer 4:1, although the low targeted MW of 5 kDa might negatively affect the activity too (**Figure** **6**). Concerning the high activity of the poly(SS‐*co*‐IA) 2:1 copolymers of 10 and 20 kDa, it appears that molecular mass does not play a significant role in inhibition. This might be attributed to the fact that the indicated copolymers with a targeted size of 10 and 20 kDa are fairly similar in their real size (Table 1). As with the previously investigated heparin‐mimetic polymers, the anticoagulant effect is significantly lower than with UFH. This offers a better safety.

### 2.7 Detecting Biocompatibility of the Poly(SS‐co‐IA) Copolymers

**Figure 7:**
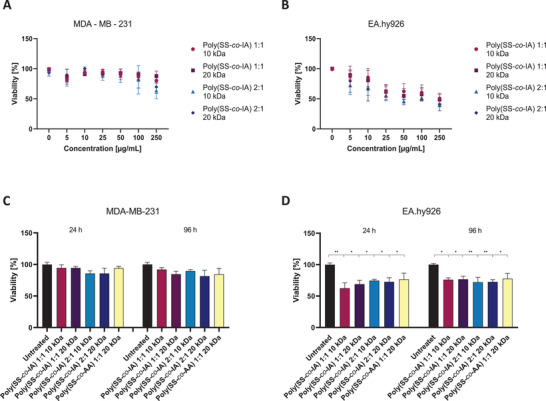
Cytotoxicity studies on tumor and endothelial cells. Investigation of the biocompatibility of the poly(SS‐*co‐*IA) copolymers on the viability of A) MDA‐MB‐231 breast cancer cells or, B) EA.hy926 endothelial cells after 72 h incubation with the indicated concentrations of polymers. Detection of the percentage of viability after a 24 or 96 h incubation time with 50 µg mL^−1^ of the indicated copolymers with C) MDA‐MB‐231 cells, or D) EA.hy926 cells.

In order to apply the poly(SS‐*co*‐IA) copolymers as potential heparin mimicking compounds in a broader biological context, their general biocompatibility has to be assured and cytotoxic effects have to be excluded. Therefore, we tried to detect critical cytotoxic concentrations of the indicated polymers in cellular in vitro systems. Therefore, we applied an MTT assay and compared the cytotoxicity in MDA‐MB‐231 breast cancer cells, used above for platelet activation, as well as in the more sensitive EA.hy926 human endothelial cells representing a physiological, non‐cancerous system. It becomes evident that the indicated polymers are well tolerated and seemingly affect the viability of the breast cancer cells only at concentrations above 250 µg mL^−1^, confirming that all data shown above at 50 µg mL^−1^ were not affected by any toxic side effects. In case of the human endothelial cells, a higher sensitivity is evident. However, the slightly reduced viability rates have to be considered in the context of the long time range of MTT (72 h incubation) compared to the much shorter incubations times used above (**Figure** **7**A,B).

Furthermore, we checked whether the indicated copolymers affect cell proliferation. Using a modified MTT assay, the proliferation rate of the two cell lines was detected in a period of 96 h in presence of 50 µg mL^−1^, representing the active concentration of the assays described above. It became evident that the proliferation rate of MDA‐MB‐231 cells is slightly diminished when compared to the untreated control cells (Figure 7C), but the reduction in proliferation appears minimal and similar across the derivatives. The endothelial cells appear to be more affected by the polymers in their initial proliferation cycles. However, while the proliferation rate in the early time points drops to a≈60% to 70%, the 96 h represent proliferation rate in the range of 75% for all polymers (Figure 7D).

Concluding, these data give rise to the assumption that the indicated poly(SS‐*co*‐IA) copolymers are well tolerated at the concentration and time ranges applied to be further investigated for their potential as heparin substitutes.

### 2.8 Poly(SS‐co‐IA) Copolymers Display Much Lower Binding Affinity to PF4, thus Possess a Lower Risk to Induce HIT II

Heparin induced thrombocytopenia type II (HIT II) is the most feared side effect of the clinical application of UFH. Up to 5% of UFH treated patients display an incidence of HIT II with varying degrees of severity. HIT II is an immunological reaction based on antibody formation against a complex formed by UFH with PF4. Undoubtedly, this is a process related to the large MW of heparin, since the incidence of HIT II is tenfold higher when using UFH compared to the application of LMWH.^[^ ^22^ ^]^ Consequently, the binding affinities of heparin derivatives to PF4 are the critical issue to trigger HIT II susceptibility. To investigate whether the poly(SS‐*co*‐IA) heparin mimetics, in light of the similar or even exceeding activities compared to UFH share this risk profile with UFH, we investigated the binding affinities of the poly(SS‐*co*‐IA) mimetics to PF4 by biosensor measurements.

Therefore, we applied a surface acoustic wave (SAW) biosensor approach. Recombinant PF4 was inserted into a sensor‐fixed model membrane in a his‐tag orientated manner^[^ ^23^ ^]^ and binding of UFH, LMWH, and the polymers to immobilized PF4 was analyzed under dynamic flow conditions (**Table** **3**).

**Table 3:** PF4 binding kinetics of poly(SS‐*co*‐IA) copolymers compared to UFH and LMWH and to poly(SS‐*co*‐AA) as well as poly(SPA‐*co*‐AA) copolymers described recently.^[^ ^17^ ^]^

Compound	K_D_ [м]	k_on_ [м^−1^ s^−1^]	k_off_ [s^−1^]
UFH	5.83 ± 3.05 × 10^−11^	3.45 ± 0.12 × 10^8^	1.71 ± 0.26 × 10^−2^
Enoxaparin	n.d.	n.d.	n.d.
Fondaparinux	n.d.	n.d.	n.d.
Poly(SS‐*co*‐IA) 1:1 10kDa	1.52 ± 0.64 × 10^−8^	2.32 ± 0.44 × 10^6^	2.70 ± 0.50 × 10^−2^
Poly(SS‐*co*‐IA) 1:1 20 kDa	1.28 ± 0.77 × 10^−8^	1.38 ± 0.32 × 10^6^	1.58 ± 0.63 × 10^−2^
Poly(SS‐*co*‐IA) 2:1 10 kDa	1.94 ± 0.98 × 10^−8^	9.59 ± 2.32 × 10^5^	1.70 ± 0.31 × 10^−2^
Poly(SS‐*co*‐IA) 2:1 20 kDa	2.88 ± 1.22 × 10^−8^	5.20 ± 1.43 × 10^5^	1.86 ± 0.35 × 10^−2^
Poly(SS) 20 kDa	9.82 ± 0.29 × 10^−8^	3.98 ± 2.53 × 10^5^	3.54 ± 2.89 × 10^−2^
Poly(SS‐*co*‐AA) 1:1 10 kDa	n.d.	n.d.	n.d.
Poly(SS‐*co*‐AA) 1:1 20 kDa	1.40 ± 1.38 × 10^−7^	7.93 ± 3.41 × 10^5^	5.34 ± 1.53 × 10^−2^
Poly(SPA‐*co*‐AA) 1:1 20 kDa	n.d.	n.d.	n.d.

While UFH displays an impressively high binding affinity to PF4 in the picomolar range (Table 3), binding of enoxaparin representing a smaller sized LMWH or of the pentasaccharide fondaparinux to PF4 could not be detected, which is reflected by much higher incidence of HIT II due to UFH than LMWH. Notably, the poly(SS‐*co*‐IA) derivatives, each at targeted MW of 10 kDa and 20 kDa, exceeding the size of UFH clearly display a more than thousand‐fold lower binding affinity to PF4 compared to UFH. This is surprising and suggests that the size of a negatively charged polymer is not the only critical parameter. Remarkably, the high value of *K*_D_ of UFH is due to very high k_on_, while dissociation rates of all compounds appear comparable.

To further address this issue of charge density, we compared similar sized polymers to poly(SS‐*co*‐IA) with lower change densities, e.g. by replacing double‐charged IA moieties by acrylic acid (AA) copolymers poly(SS‐*co*‐AA), or by applying poly(SS) 20 kDa polymer.^[^ ^15^ ^]^ Both polymers display roughly ten‐fold lower affinities to PF4 than the poly(SS‐*co*‐IA) derivatives indicating a certain impact of charge for affecting PF4 binding, which however cannot solely explain the outstanding affinity of UFH.

Nevertheless, these data indicate the promise of the novel copolymers with respect to a much lower HIT II susceptibility when compared to UFH.

## 3 Conclusion

Here we introduce a promising new series of structurally well‐defined and tunable heparin‐mimicking synthetic copolymers, poly(SS‐*co*‐IA), which successfully interferes with different processes in the simulated hematogenous metastasis of tumors. The indicated polymers possess excellent anticoagulant activities, partly outperforming LMWH, and on that basis they target different aspects of tumor cell‐induced platelet activation. Beyond anticoagulation, the tunable size and charge density of the polymers is beneficial for optimizing targeted activities, such as blockade of enzymatic activity of HPSE. Although various studies are needed focusing on the potential in vivo performance of these heparin mimetics, with respect to bioactivity, biocompatibility and pharmacokinetics for a perspective substitution of heparin natural products in therapy, these polymers are already now valuable tools to elucidate the molecular activities of heparin in oncology, thus following an important translational path from a molecular understanding into clinical oncology.

## 4 Experimental Section

### General

4, 4′‐Azobis‐(4‐cyanovaleric acid) (≥75%) (ACVA), itaconic acid (IA), potassium 3‐sulfopropyl methacrylate (SPMA), sodium 2‐acrylamido‐2‐methyl‐1‐propane sulfonate (50 wt.% in H_2_O) (AMPS), sodium 4‐styrenesulfonate (SS), and 1, 3, 5‐trioxane were purchased from Sigma Aldrich (Australia). The RAFT agent ((((1‐carboxyethyl)thio)carbonothioyl)thio)propanoic acid (BM1429), commercially available from Boron Molecular Pty Ltd, was kindly provided by the Commonwealth Scientific and Industrial Research Organization (CSIRO), Clayton, Melbourne. All reactions were carried out in a microwave reactor (CEM Discover S system, CEM Corporation, USA), and monomer conversions were determined via ^1^H NMR spectroscopy on a Bruker Avance 300 or 400 NMR spectrometer. Polymers were dissolved in D_2_O and 1, 3, 5‐trioxane served as the internal standard (δ 5.12). Molecular weight and dispersities (*M*_w_/*M*_n_, *Ð*) were determined by gel permeation chromatography (GPC), see Table 1 and Supporting Information.

### Polymer Synthesis

All copolymers were prepared by similar methods following the previously published protocol.^[^ ^15^, ^16^ ^]^ A typical procedure for the preparation of the 1:1 (*M*_n (calc)_ = 8.4 kDa) copolymer poly(SS‐*co*‐IA) was as follows: to a 10 mL microwave reaction vial equipped with a magnetic stirrer bar ACVA (2.0 mg, 0.007 mmol), BM1429 (9.08 mg, 0.036 mmol), SS (206.2 mg, 1.0 mmol), IA (130 mg, 1.0 mmol), 1,3,5‐trioxane (10.0 mg, 0.11 mmol; internal reference for NMR analysis) and deionized water (1.7 mL) were added. The mixture was stirred to obtain a homogenous solution. The solution was then purged with nitrogen for 10 min, and an aliquot of the solution was then diluted with D_2_O to obtain the initial (t = 0) ^1^H NMR spectrum. The reaction mixture was heated in the microwave reactor at 85 °C for 4 h. After completion, an aliquot of the solution was diluted in D_2_O to obtain the final ^1^H NMR spectrum for calculating the monomer conversion. Finally, the solution was concentrated at 40 °C using a rotary evaporator and the product was subsequently isolated by precipitation from acetone followed by drying in an oven at 50 °C for ≈48 h. The product yield was 130 mg (38%).

### Monomer conversion and molecular weight determination by ^1^H NMR spectroscopy

The theoretical molecular weights of all synthesized copolymers were determined using the following equation after calculating the monomer conversion from the ^1^H NMR spectra:

(1) $$\begin{array}{c} {{\left[M_{n}\right]}_{t}}_{\left(\mathrm{calc}\right)}=\frac{\left({\left[M_{1}\right]}_{0}-{\left[M_{1}\right]}_{t}\right)\cdot MW_{1}+\left({\left[M_{2}\right]}_{0}-{\left[M_{2}\right]}_{t}\right)\cdot MW_{2}}{{\left[\mathrm{RAFT}\right]}_{0}}+M_{\mathrm{RAFT}} \end{array}$$

where [M_n_]_t (calc)_ = theoretical molecular weight; [M]_0_ = concentration of monomer at time 0; [M]_t_ = concentration of monomer at time t; MW = Molecular weight of the monomer; [RAFT]_0_ = concentration of the RAFT agent at time 0.

### Gel Permeation Chromatography

GPC analyses were carried out using a Nexera‐I LC2040C 3D GPC/SEC (Shimadzu, Australia) equipped with a RID‐20A Refractive Index Detector and operating at a flow rate of 1 mL min^−1^. The columns (PL aquagel‐OH MIXED‐H, 300 × 7.5 mm, PL aquagel‐OH Guard 8 µm, 50 × 7.5 mm) were maintained at 40 °C and the mobile phase was 47 mM NaNO_3_ (aq) with 3 mM NaN_3_ (aq). The samples were dissolved over 16 h in the mobile phase solution at a concentration of 2 to 4.4 mg mL^−1^ and filtered through a 13 mm 0.45 µm membrane filter (PVDF) before injection (10 µL). The sample holder was maintained at 15 °C. The molecular weights were referenced against PEO narrow standards (Agilent).

### Heparanase Inhibition Assay

Heparanase (HPSE) enzymatic activity was determined using Homogeneous Time Resolved Fluorescence (HTRF), a Time‐Resolved Fluorescence Resonance Energy Transfer (TR‐FRET–based) assay (Cisbio Bioassays S.A.S., Codolet, France). All compounds were dissolved and diluted to different concentrations using sample buffer (50 mм Tris‐HCl pH 7.4, 0.15 м NaCl, 0.1% protease free BSA, 0.1% CHAPS). 3 µL of compound solution or sample buffer (as a control) and 7 µL semi‐purified HPSE of solution or sample buffer (as a blank) were added into 384‐well low volume microplate. After 10 min pre‐incubation at 37°, an enzyme reaction was initiated by adding 5 µL Biotin‐HS‐Eu (K) (4.2 ng in 0.2 M acetate buffer pH 5.5) to each well, and the plate was incubated for 30 min at 37 °C. To stop the enzyme reaction and detect the remaining substrate, 5 µL streptavidin‐d2 (12 ng in 62.5 mM Hepes pH 7.4, 0.8 M KF, 0.1% protease free BSA, 2 mg mL^−1^ heparin) were added into each well. After 15 min incubation at RT, the HTRF signal was measured by FLUOstar OMEGA reader (BMG Labtech) using the following setup: excitation filter 337 nm, emission filters 620 nm and 665 nm.

### Cell Lines

MDA‐MB‐231 human breast cancer cells (ATCC HTB‐26) were cultured in Dulbecco`s Modified Eagle Medium (DMEM) containing 4.5 g L^−1^ D‐glucose, L‐glutamine and pyruvate (Gibco, Grand Island, NE, USA) with added 100 U/mL penicillin and 100 µg mL^−1^ streptomycin (PAN Biotech, Germany). Human endothelial cell line EA.hy926 was cultured in a DMEM – low glucose medium (Sigma‐Aldrich Chemie GmbH, Taufkirchen, Germany). To prepare the ready‐to‐use medium, 10 g (1 vial) of the lyophilized medium was dissolved in one liter of sterile Millipore water. The medium was supplemented with 3.7 g L^−1^ sodium hydrogen carbonate, 100 mL FKS and 10 mL penicillin‐streptomycin solution in concentrations of 10000 IU mL^−1^ or 10 mg mL^−1^, respectively.

### Platelet Preparation

The platelet‐rich plasma (PRP) was provided by healthy donors obtained from the Institute for Experimental Hematology and Transfusion Medicine, University Hospital Bonn. The platelets were centrifuged at 670 × g for 10 min at RT and the resulting platelet pellet was resuspended using platelet buffer (10 mм HEPES, 136.7 mм NaCl, 2.6 mм KCl, 1 mм MgCl_2_, 13.8 mм NaHCO_3_, 0.36 mм NaHPO_4_ and 5.5 mм glucose) to a final concentration of 4 × 10^8^ plts/mL. The platelet suspension was supplemented with a 1 mм CaCl_2_ solution, mixed with test compounds at the specified concentrations and incubated for 30 min before the respective assays were started. The commercial anticoagulants were used at simulated therapeutic concentrations, e.g. UFH (Ratiopharm GmbH, Ulm, Germany), and enoxaparin (Venipharm, Hamburg, Germany) at 1 U/mL, and fondaparinux (Aspen Pharma Trading Ltd, Dublin, Ireland) at 0.78 µg mL^−1^, respectively. Synthetic heparin mimetic polymers were used at 50 µg mL^−1^. The thrombin generation assay was run using twelvefold higher concentrations.

### Light Transmission Aggregometry

Tumor cell‐induced platelet aggregation was performed using the APACT 4004 (Haemochrom Diagnostica GmbH, Essen, Germany) light transmission aggregometer. Platelets were prepared as previously described and continuously heated at 37 °C and stirred at 1000 rpm in cuvettes to record the kinetics of aggregation. As activation factors 41.25 µм TRAP‐6 (Cayman Chemical, Ann Arbor, MI, USA) or 1 × 10^4^ MDA‐MB‐231 cells were used. Data for 0% aggregation were considered when measuring untreated, non‐activated platelets, 100% aggregation was equivalent to using control platelet buffer without platelets.

### ATP Assay

To quantify platelet activation and subsequent ATP release from the granules, a luciferase‐based ATP determination kit (Thermo Fisher scientific) was used in a 96 well format according to the manufacturer's instructions. Platelets were prepared as described above and activated for 12 min with 41.25 µм TRAP‐6 or 1 × 10^4^ MDA‐MB cells. Samples were measured using a Fluostar optima plate reader (BMG Labtech, Ortenberg, Germany). Data were normalized to the ATP release of the TRAP‐6 sample.

### Thrombin Generation Assay

Tumor cell‐induced thrombin generation in human platelet concentrate was investigated using a TECHNOTHROMBIN assay kit (Technoclone, Vienna, Austria), which is based on thrombin‐specific cleavage of a fluorogenic substrate, Z‐Gly‐Gly‐Arg‐AMC. To inhibit the intrinsic thrombin generation pathway, corn trypsin inhibitor (Santa Cruz Biotechnology, USA) was added to platelet‐rich plasma at a concentration of 50 µg mL^−1^. The PRP was then incubated with the substances for 30 min. Afterward, the samples were transferred to a 96‐well plate, MDA‐MB‐231 cells were added to a final concentration of 1 × 10^4^ cells per mL. The integrity of the PRP was checked by activation with the recombinant tissue factor reagent Technothrombin TGA RC high (Technoclone GmbH, Vienna, Austria), while the negative control was determined by adding DPBS to an untreated sample. The samples were recalibrated by adding the reagent Technothrombin TGA SUB (Technoclone GmbH, Vienna, Austria) and shortly after the addition, the kinetics of thrombin generation were continuously measured using a Tecan Spark microplate reader (Tecan Group, Männedorf, Switzerland). The fluorescence signals were converted to thrombin concentrations using the analysis software provided by Technoclone GmbH.

### Activated Partial Thromboplastin Time (APTT) Assay

The APTT assays were conducted following the reported protocol.^[^ ^24^ ^]^ Briefly, the STA‐PTT reagent was reconstituted in 5.0 mL of distilled water according to the manufacturer's instructions. Similarly, the two individual vials for system control N + P were also reconstituted in 1.0 mL of distilled water. All reagents were then placed onto the loading drawer of the analyzer, STA‐R hemostasis analyzer (Diagnostica Stago France). All test polymers were dissolved in distilled water to a final concentration of 2 mg mL^−1^. Individual polymer solution was then spiked into pooled normal plasma prior to the initiation of the APTT assay depending on the target concentration. Varying concentrations of UFH and LMWH were also prepared as the standards. The loaded plasma (50 µL) was mixed with APTT (50 µL) reagents and incubated for 4 min. After incubation, coagulation was initiated by adding CaCl_2_ (50 µL), and then the time required for clot formation was recorded in seconds. The STA‐R analyzer performed the assay automatically where all these conditions were installed. Clot detection by the STA‐R involves an electromagnetic−mechanical system. The oscillation of a steel ball within the cuvette containing the plasma is monitored. When the oscillation of the steel ball is stopped by clot formation, the sensor registers the time in seconds.

### Cytotoxicity Assay

Potential cell cytotoxicity of indicated mimetics was investigated using MTT 3‐(4,5‐dimethylthiazol‐2‐yl)‐2,5‐diphenyltetrazolium bromide assay (BioChemica, Applichem GmbH, Darmstadt, Germany). A total of 5000 MDA‐MB‐231 cells or 8000 EA.hy926 cells were seeded as triplicates at a total volume of 90 µL per well of 96‐well plates (Starlab International GmbH, Hamburg, Germany). After 24 h of incubation, the cells are treated with the compounds (5 to 250 µg mL^−1^). After an incubation period of 72 h, the MTT solution was added (5 mg mL^−1^, 20 µL) and incubated for 1 h at 37 °C and 5% CO_2_. After removal of the supernatant, formed formazan was dissolved in 100 µL DMSO (Carl Roth GmbH, Karlsruhe, Germany). The plate was measured at 570 nm with a background correction of 690 nm using a plate reader (Thermo Multiscan EX, Thermo, Schwerte, Germany). The data was normalized to DPBS as 100% viability.

### Proliferation

The proliferation assay was performed based on the cytotoxicity assay. The cell suspensions were seeded into the wells and treated with a final concentration of 50 µg mL^−1^ of the indicated polymers. The plates were incubated for 24, 72, and 96 h at 37 °C and 5% CO_2_. MTT measurements was similar as described above. The data were normalized to the untreated sample as 100% viability.

### Surface Acoustic Wave Biosensor

The binding affinity between heparin or polymer mimetics and PF4 was determined using a surface acoustic wave‐based biosensor sam5 system (SAW instruments, Bonn, Germany), as described before.^[^ ^17^ ^]^ For this technology, the target peptide carrying a his‐tag was bound to the surface of a sensor‐attached model membrane containing a nickel complexed lipid DOGS‐NTA (1,2‐dioleoyl‐sn‐glycero‐3‐[(N‐(5‐amino‐1‐carboxypentyl) iminodiacetic acid) succinyl]), obtained from Merck KgaA, Darmstadt, Germany.^[^ ^23^ ^]^ For the measurement, his‐tagged PF‐4 (2.5 µg) (Bon Opus Biosciences, Millburn NJ, USA) was injected into the measurement device at a flow rate of 20 µL mi^−1^n allowing immobilization of the protein at the sensor surface. Afterward, the system was equilibrated at a flow rate of 40 µL mi^−1^n until the phase shift has reached a constant value. For the detection of binding events between the compounds and PF‐4, dilution series between 1.04 × 10^−9^ to 5.2 × 10^−7^ м of UFH, LMWH or polymer mimetics were prepared. The measurements were performed at room temperature with a continuous buffer flow of 40 µL mi^−1^n. After each compound injection, a rinse injection with 4 м NaCl solution was performed to remove the heparins or polymers from the immobilized protein. After this reconstitution of the protein, the next higher concentration of the test substances was measured. K*_D_*, k_on_, and k_off_ values were extracted from the binding curves using the SamBlue FitMaster software.

### Statistical Analysis

Data represent means ± SD and originate from at least three independent biological replicates (*N* = 3). Statistical analysis was performed using one‐way ANOVA followed by Dunnett's test as a post‐hoc test. Statistical significance is marked with an asterisk (* = *p* < 0.05; ** = *p* < 0.01; *** = *p* < 0.001; **** = *p* < 0.0001).

## Conflict of Interest

The authors declare no conflict of interest.

## Author Contributions

The manuscript was written through contributions of all authors. All authors have given approval to the final version of the manuscript.

## Supporting information



Supporting Information

## Data Availability

The data that support the findings of this study are available from the corresponding author upon reasonable request.
