# Supplementary material for: A Novel Series of Synthetic Heparin‐Mimetics–Itaconic Acid‐Containing Copolymers for Targeting Tumor Cell Coagulability and Metastasis
Source: Macromol Biosci. 2025 Apr 7;25(7):2400633. doi: 10.1002/mabi.202400633 (PMC12259396; doi:10.1002/mabi.202400633)
Supplement: Supplementary file 1 — Supporting Information [file MABI-25-2400633-s001.docx]

# SUPPORTING INFORMATION

# A novel series of synthetic heparin-mimetics – Itaconic acid-containing copolymers for targeting tumor cell coagulability and metastasis

Katrin Nekipelov^#^, Abdullah Al Nahain ^🗶^, Sven Otto^#^, Yongbin Xu^🞎^, Jin-Ping Li^🞎^, Natasha Letunica,^§^ Simon Collett, ^§^ Chantal Attard, ^§^ Paul Monagle, ^§^ George Vamvounis,^ǂ^ John Tsanaktsidis,^◊^ Vito Ferro^🗶^ and Gerd Bendas^#^

^#^Pharmaceutical Institute, Pharmaceutical and Cell Biological Chemistry, University of Bonn, 53121Bonn, Germany

^🗶^School of Chemistry and Molecular Biosciences, The University of Queensland, Brisbane, QLD 4072, Australia

^🞎^Department of Medical Biochemistry and Microbiology, The Biomedical Center, University of Uppsala, 75123 Uppsala, Sweden

^§^Haematology Research, Murdoch Children's Research Institute, Parkville, Victoria 3052; Department of Paediatrics, The University of Melbourne, Parkville, Victoria 3052; Department of Clinical Haematology, Royal Children's Hospital, Parkville, Victoria 3052, Australia

^ǂ^College of Science and Engineering, James Cook University, Townsville, QLD 4811, Australia

^◊^CSIRO Manufacturing, Research Way, Clayton, Victoria 3168, Australia

**Contents**

Page

GPC chromatograms (Fig. S1-10) S3-S7

^1^H NMR spectra (Fig. S11) S8

Aggregation Assays (Fig.12) S9

Thrombin generation Assay (Fig.13) S10

**Figure S1**. GPC chromatogram of poly(SS-*co*-IA) 1:1 polymers

**Figure S2**. GPC chromatogram of poly(SS-*co*-IA) 2:1 polymers

**Figure S3**. GPC chromatogram of poly(SS-*co*-IA) 4:1 5 kDa polymer

**Figure S4**. GPC chromatogram of poly(SS-*co*-IA) 1:4 10 kDa polymer

**Figure S5**. GPC chromatogram of poly(SS-*co*-IA) 1:2 20 kDa polymer

**Figure S6**. GPC chromatogram of poly(AMPS-*co*-IA) 2:1 polymers

**Figure S7**. GPC chromatogram of poly(AMPS-*co*-IA) 4:1 polymers

**Figure S8**. GPC chromatogram of poly(SPMA-*co*-IA) 1:1 polymers

**Figure S9**. GPC chromatogram of poly(SPMA-*co*-IA) 2:1 polymers

**Figure S10**. GPC chromatogram of poly(SPMA-*co*-IA) 4:1 polymers

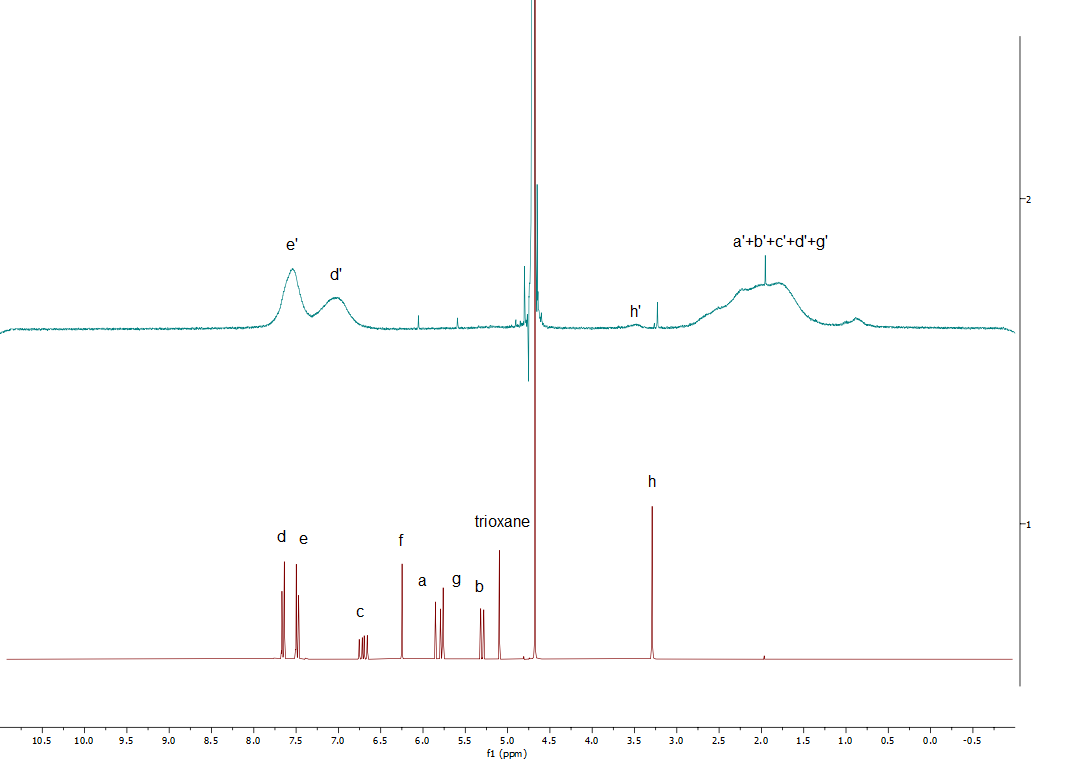


a’+b’+c’+f’+g’+h’

Figure S11. Representative ^1^H NMR spectra (400 MHz) for the 1:1 mixture of SS and IA monomers (bottom) prior to initiation of polymerization and the copolymer poly(SS-*co*-IA) 1:1 (top) in D_2_O.

**A**

**B**

**C**

**Figure S12**. Tumor cell-induced platelet aggregation in presence of AMPS and SPMA copolymers

**A**

**B**

**Figure S13**. Inhibition of thrombin generation with further SPMA polymers (2:1 and 4:1 copolymers).
